# Traditionally Used *Lathyrus* Species: Phytochemical Composition, Antioxidant Activity, Enzyme Inhibitory Properties, Cytotoxic Effects, and *in silico* Studies of *L. czeczottianus* and *L. nissolia*

**DOI:** 10.3389/fphar.2017.00083

**Published:** 2017-02-27

**Authors:** Eulogio J. Llorent-Martínez, Gokhan Zengin, María L. Fernández-de Córdova, Onur Bender, Arzu Atalay, Ramazan Ceylan, Adriano Mollica, Andrei Mocan, Sengul Uysal, Gokalp O. Guler, Abdurrahman Aktumsek

**Affiliations:** ^1^Regional Institute for Applied Chemistry Research, University of Castilla-La ManchaCiudad Real, Spain; ^2^Department of Biology, Science Faculty, Selcuk UniversityKonya, Turkey; ^3^Department of Physical and Analytical Chemistry, University of JaénJaén, Spain; ^4^Biotechnology Institute, Ankara UniversityAnkara, Turkey; ^5^Department of Pharmacy, University “G. d'Annunzio” of Chieti-PescaraChieti, Italy; ^6^Department of Pharmaceutical Botany, “Iuliu Hatieganu” University of Medicine and PharmacyCluj-Napoca, Romania; ^7^Institute for Life Sciences, University of Agricultural Sciences and Veterinary Medicine Cluj-NapocaCluj-Napoca, Romania; ^8^Department of Biological Education, Ahmet Kelesoglu Education Faculty, Necmettin Erbakan UniversityKonya, Turkey

**Keywords:** *Lathyrus*, flavonoid, antioxidant, cytotoxic, molecular docking, bioactive formulations

## Abstract

Members of the genus *Lathyrus* are used as food and as traditional medicines. In order to find new sources of biologically-active compounds, chemical and biological profiles of two *Lathyrus* species (*L. czeczottianus* and *L. nissolia)* were investigated. Chemical profiles were evaluated by HPLC-ESI-MS^n^, as well as by their total phenolic and flavonoid contents. In addition, antioxidant, enzyme inhibitory, and cytotoxic effects were also investigated. Antioxidant properties were tested by using different assays (DPPH, ABTS, CUPRAC, FRAP, phosphomolybdenum, and metal chelation). Cholinesterases (AChE and BChE), tyrosinase, α-amylase, and α-glucosidase were used to evaluate enzyme inhibitory effects. Moreover, vitexin (apigenin-8-C-glucoside) and 5-O-caffeoylquinic acid were further subjected to molecular docking experiments to provide insights about their interactions at molecular level with the tested enzymes. *In vitro* cytotoxic effects were examined against human embryonic kidney cells (HEK293) by using iCELLigence real time cell analysis system. Generally, *L. czeczottianus* exhibited stronger antioxidant properties than *L. nissolia*. However, *L. nissolia* had remarkable enzyme inhibitory effects against cholinesterase, amylase and glucosidase. HPLC-ESI-MS^n^ analysis revealed that flavonoids were major components in these extracts. On the basis of these results, *Lathyrus* extracts were rich in biologically active components; thus, these species could be utilized to design new phytopharmaceutical and nutraceutical formulations.

## Introduction

With recent advances in science, the therapeutic properties of plants have gained importance all over the world, due to their pharmacological activities (antioxidant, anti-cancer, anti-inflammatory, antimicrobials, etc.) and nutraceuticals properties (Krishnaiah et al., 2011). Many reports have focused on benefits of phytochemicals from plants and their health-promoting effects. Among the various phytochemicals with biological properties, phenolic compounds are responsible for the above-mentioned biological activities (Shahidi and Ambigaipalan, 2015). Actually, in global communities increasingly concerned on health and nutrition, these products are emerging as valuable alternatives for replacing synthetic drugs. On the basis of the aforementioned concerns, new plant-derived products or phytomedicines are considered as prospective materials.

The genus *Lathyrus*, which belongs to Fabaceae, is represented by more than 200 species worldwide (Alkin et al., 1986). This genus consists of 75 taxa, of which 18 species are endemic in Turkish Flora (Genç, 2009). Like most members of Fabaceae, *Lathyrus* genus presents different uses, such as alimentary, agricultural, industrial, ornamental, and in traditional medicine (Patto and Rubiales, 2014). For instance, some *Lathyrus* species such as *L. hirsutus* L., *L. cicera* L., *L. odoratus* L., *L. ochrus* L. (DC.), *L. sylvestris* L., and *L. palustris* L., which have high nutrient quality, are consumed as food (for both humans and animals) (Chavan et al., 2001). Especially, roots of *L. odoratus* are used as food in The East Anatolian Region of Turkey. Again, *L. odoratus* and *L. sativus* L. are used for agricultural processes in Turkey. Moreover, *Lathyrus* species have also importance in folk medicine for several purposes such as analgesic (seed of *L. sativus*), anti-inflammatory (aerial parts of *L. cicera*), and anti-rheumatism (leaf of *L. rotundifolius* Willd. subsp. *miniatus* (Bieb. ex Stev.) Davis) in Turkey (Altundag and Ozturk, 2011). However, up-to-date information about *L. czeczottianus* Bässler and *L. nissolia* L. chemical composition or biological characterization is scarce. Considering the importance of different *Lathyrus* species, as previously mentioned, the general aim of this research is to improve the knowledge about less-known *Lathyrus* plants. In this direction, this study was designed to investigate: (i) the antioxidant activity of the methanolic extracts of *L. czeczottianus* and *L. nissolia* using various *in vitro* biochemical assays, (ii) the enzyme inhibitory potentials (anti-cholinesterase, anti-amylase, anti-glucosidase, and anti-tyrosinase), (iii) the cytotoxicity in human embryonic kidney cells using the real time cellular impedance technology, (iv) the characterization of phytochemical compounds in the methanolic extracts by HPLC-ESI-MS, (v) and the components-enzymes interactions with *in-silico* techniques. Thus, our present study may contribute to offer new perspectives on the biological properties and phytochemical profile of the genus *Lathyrus*.

## Experimental

### Chemicals and reagents

All reagents and standards were of analytical reagent grade unless stated otherwise. Kaempferol (≥97%), luteolin (≥98%), quercetin (≥95%) and rutin (≥95%) were purchased from Sigma-Aldrich (St. Louis, MO, USA) and 200 mg/L stock solutions were prepared in ethanol (HPLC grade; Sigma). LC–MS grade acetonitrile (CH_3_CN, 99%) (LabScan; Dublin, Ireland) and ultrapure water (Milli-Q Waters purification system; Millipore; Milford, MA, USA) were used for the HPLC-MS analyses. Folin–Ciocalteu's reagent and methanol were purchased from Merck (Darmstadt, Germany). 2,2-Diphenyl-1-picrylhydrazyl (DPPH), ABTS radical cation (2,2′-azino-bis(3-ethylbenzothiazoline)-6-sulphonic acid), DTNB (5,5-dithio-bis(2-nitrobenzoic) acid) and AChE (acetylcholinesterase (Electric ell acetylcholinesterase, Type-VI-S, EC 3.1.1.7), BChE (butyrylcholinesterase (horse serum butyrylcholinesterase, EC 3.1.1.8), L-DOPA (3,4-dihydroxy-L-phenylalanine), tyrosinase, acetylthiocholine iodide (ATCI) and butyrylthiocholine chloride (BTCl) were purchased from Sigma Chemical Co. All other chemicals and solvents were of analytical grade.

### Plant material and extraction procedure

*Lathyrus* species were collected at flowering stage and corresponding information and localities are explained below. Taxonomic identification of the plant materials was confirmed by senior taxonomist Dr. Murad Aydın Sanda [Selcuk University, Science Faculty, Department of Biology (Botany)] based on Flora of Turkey (Davis, 1970) and A checklist of the Flora of Turkey (Vascular Plants) (Güner et al., 2012). Voucher specimens were deposited at the KNYA Herbarium of Department of Biology, Selcuk University, Konya-Turkey.

1. *Lathyrus czeczottianus* Bässler: Locality: Ankara, Cubuk, around Karagol, forest clearings, Turkey. Date: 25/06/2013. Collector: Ramazan Ceylan and Gokhan Zengin. Life cycle stage of the plant: flowering. Plant part: aerial parts. Family: Fabaceae.
2. *Lathyrus nissolia* L.: Locality: Ankara, Cubuk, around Karagol, forest clearings, Turkey. Date: 13/06/2013. Collector: Ramazan Ceylan and Gokhan Zengin. Life cycle stage of the plant: flowering. Plant part: aerial parts. Family: Fabaceae.

The plant materials were dried at room temperature. The dried aerial parts were ground to a fine powder using a laboratory mill. To obtain methanolic extracts, the air-dried aerial parts (10 g) were macerated with 200 mL of methanol at room temperature (25°C ± 1°C) for 24 h. The extracts were concentrated under vacuum at 40°C by using a rotary evaporator and stored at + 4°C in dark until use.

### Chromatographic conditions

The HPLC system consisted of a vacuum degasser, an autosampler and a binary pump (Agilent Series 1100, Agilent Technologies, Santa Clara, CA, USA) equipped with a reversed phase Kinetex core-shell C_18_ analytical column of 50 × 2.1 mm and 2.6 μm particle size (Phenomenex, Torrance, CA, USA). A C_18_ Security Guard Ultra cartridge (Phenomenex) of 2.1 mm i.d. was placed before the analytical column. The best separation was achieved by using a mobile phase consisting of acetonitrile (A) and water-formic acid (100:0.1, v/v) (B). The following gradient program was used: 10% A (0 min), 25% A (10–20 min), 50% A (40 min), 100% A (42–47 min), and 10% A (49 min). The mobile phase flow rate was 0.4 mL min^−1^. After filtration through 0.45 μm PTFE membrane filters, 10 μL of each extract was injected.

The HPLC system was connected to an ion trap mass spectrometer (Esquire 6,000, Bruker Daltonics, Billerica, MA, USA) equipped with an electrospray interface operating in negative ion mode. The scan range was set at m/z 100–1200 with a speed of 13,000 Da/s. The ESI conditions were as follows: drying gas (N_2_) flow rate and temperature, 10 mL/min and 365°C; nebulizer gas (N_2_) pressure, 50 psi; capillary voltage, 4,500 V; capillary exit voltage, −117.3 V. The acquisition of MS^n^ data was made in auto MS^n^ mode, with isolation width of 4.0 m/z, and fragmentation amplitude of 0.6 V (MS^n^ up to MS^4^). Esquire control software was used for the data acquisition and Data Analysis for processing.

### Antioxidant and enzyme inhibitory assays

The total phenolic content was determined by Folin-Ciocâlteu method and expressed as gallic acid equivalents (GAEs/g extract), while total flavonoid content was determined by AlCl_3_ method and expressed as rutin equivalents (REs/g extract) (Vlase et al., 2014; Zengin et al., 2016). The antioxidant capacity was investigated by different assays: radical scavenging assays (DPPH and ABTS), reducing power (CUPRAC and FRAP), phosphomolybdenum, and metal chelating (ferrozine method) (Dezsi et al., 2015; Toma et al., 2015; Mocan et al., 2017). Enzyme inhibitory activities were detected against cholinesterase, α-amylase and α-glucosidase in 96-well plates. The experimental procedures for antioxidant and enzyme inhibitory assays were previously described by Mocan et al. (2016b) and Zengin et al. (2016). The antioxidant and enzyme inhibitory effects were evaluated by IC_50_ (%50 of free radical/ enzyme inhibition) and EC_50_ (the effective concentration at which the absorbance was 0.5 in CUPRAC, FRAP and phosphomolybdenum assays) values. The extracts were evaluated at 0.5–5 mg/mL in the antioxidant and enzyme inhibitory assays. Trolox (0.05–0.5 mg/mL), EDTA (0.01–0.04 mg/mL), galantamine (1–5 μg/mL), kojic acid (0.1–0.4 mg/mL) and acarbose (1–4 mg/mL) were used as positive controls in these assays. The calibration curves were used to calculate IC_50_ and EC_50_ values for each extract and reference compounds.

### Molecular modeling

#### Receptors preparation

All the crystallographic enzyme structures have been downloaded from the Protein Databank RCSB PDB (Berman et al., 2000): acetylcholinesterase (pdb:4X3C) (Pesaresi and Lamba) in complex with tacrine-nicotinamide hybrid inhibitor, butyrilcholinesterase (pdb:4BDS) (Nachon et al., 2013) in complex with tacrine, amylase (pdb:1VAH) (Zhuo et al., 2004) in complex with r-nitrophenyl-α-D-maltoside, glucosidase (pdb:3AXI) (Yamamoto et al., 2011) in complex with maltose, and tyrosinase (pdb:2Y9X) (Ismaya et al., 2011) in complex with tropolone. Waters, inhibitors and all the other molecules present in the pdb files were removed by using Pymol (DeLano, 2002) and the proteins alone were neutralized at pH 7.4 by Epik suit implemented in Maestro 9.2 suite (Maestro, 2011). Seleno-cysteines and seleno-methionines, if present, were converted respectively to cysteines and methionines. All the missing fragments and other errors present in the crystal structures were automatically solved by the Wizard Protein Preparation implemented in Maestro 9.2 suite (Maestro, 2011).

#### Ligands preparation

**Scheme 1:**
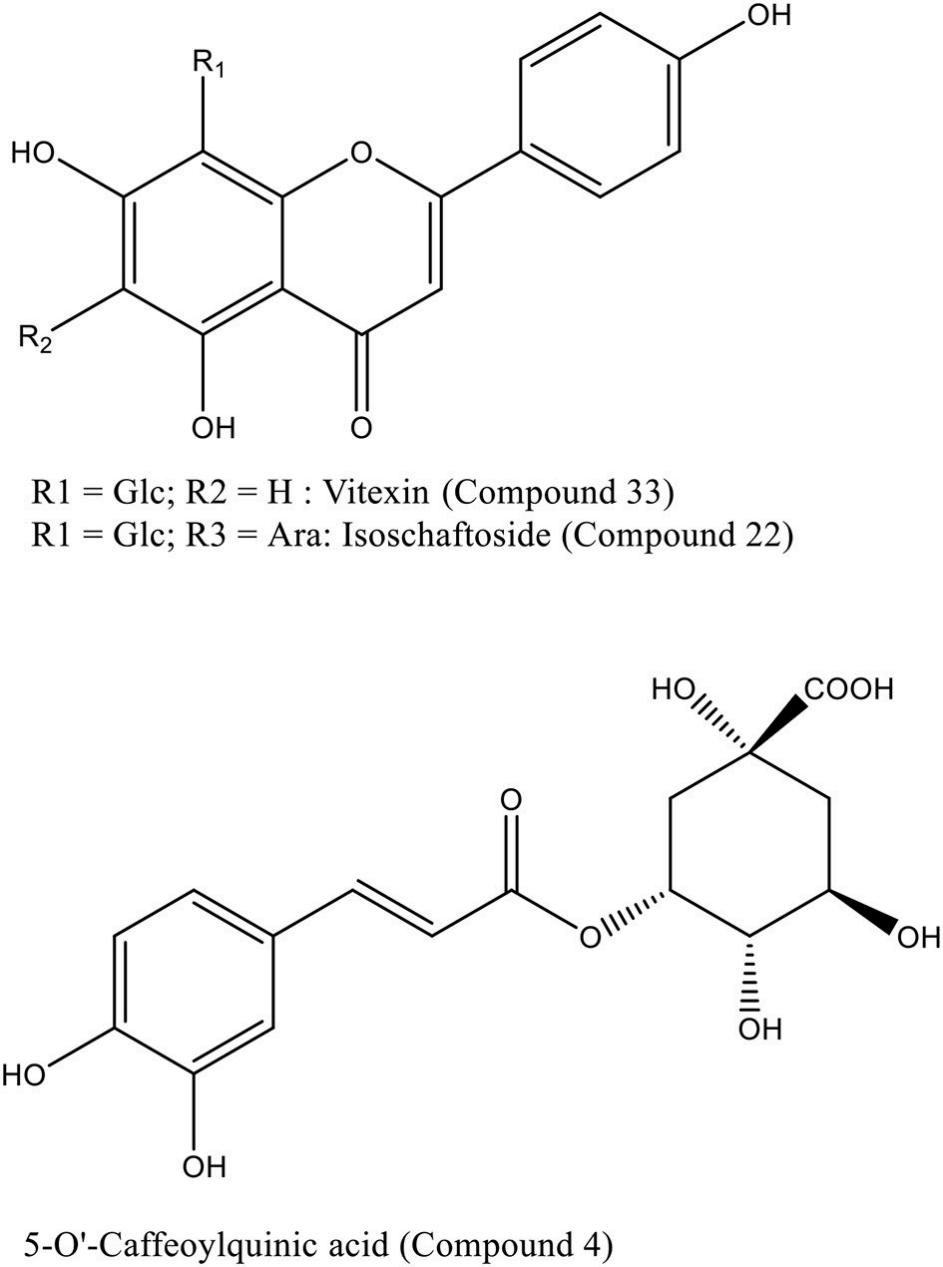
**Chemical structures of compounds Vitexin, Isoschaftoside, and 5-O-caffeoylquinic acid employed for docking experiments**.

5-O-caffeoylquinic acid (5-O-CQA, compound **4**), isoschaftoside (compound **22**) and vitexin (compound **33**) were selected as representative compounds to carry out molecular docking studies, as these compounds were present in *L. czeczottianus* and *L. nissolia* extracts. The chemical structures, reported in Scheme 1, have been downloaded from Zinc databases (Irwin et al., 2012) and used for molecular modeling experiments. The ligands were prepared by the LigPrep tool embedded in Maestro 9.2, neutralized at pH 7.4 by Epik and minimized (Shelley et al., 2007).

#### Molecular docking

Dockings of vitexin (**33**), isoschaftoside (**22**) and 5-O-CQA (**4**) have been performed for each selected enzyme employed for the *in vitro* enzymatic inhibition tests in this work. Glide (Friesner et al., 2006) has been employed for the docking calculations by using all the eXtra Precision scoring function for all the enzymes, with the exception of the docking to tyrosinase; in this case, Gold 6.0 (Jones et al., 1997) was used with the scoring function Gold Score, which has been previously found to be more suitable to produce reliable poses on metal containing enzymes (Mocan et al., 2016b). In both cases, the binding pocket was determined automatically by centering the grid on the crystallographic inhibitor, extended in a radius of 10 Angstroms from the center. The best pose for each compound docked to the selected enzymes was the best ranked among the 200 generated.

### Cytotoxicity assay

#### Cell culture

Human embryonic kidney cells (HEK-293) were obtained from Leibniz-Institute DSMZ (German Collection of Microorganisms and Cell Cultures, Braunschweig, Germany) and were cultured in Dulbecco's Modified Eagle's Medium (DMEM) (Lonza, Basel, Switzerland) containing 10% heat inactivated fetal bovine serum, 1% penicillin/streptomycin 100 U mL^−1^ and 1% L-glutamine 200 mM. Cells were maintained at 37°C in a humidified atmosphere of 5% CO_2_.

#### Cytotoxicity assay with the iCELLigence system

Cytotoxic activity was performed by using the iCELLigence real time cell analysis technology (RTCA) (ACEA Biosciences, San Diego, CA, USA) as described previously (Lazarova et al., 2015). In brief, HEK-293 cells were seeded in E-Plate L8 at a density of 5 × 10^4^ cells/well in 300 μL of growth medium. The system was set to take measurements every 30 min for 72 h. After 24 h, when the cells were in exponential phase, they were treated with 300 μL medium containing 2,000, 1,000, 500, 250, 125, 62.5 μg/mL of *L. czeczottianus* or *L. nissolia* methanolic extracts prepared in DMEM containing 0.1% dimethyl sulfoxide (DMSO). Cells were treated with 0.1% of DMSO as negative control and 5% DMSO as positive control in all experiments. Data analysis was performed using RTCA iCELLigence software and IC_50_ values (μL/mL) were automatically calculated based on the final readings obtained at the 72 h time point. All experiments were repeated three times.

### Morphological evaluation of the cells

Cells were plated at a density of 5 × 10^5^ cells/well in 6-well plate with 2 mL growth medium. Twenty-four hours after seeding, the medium was removed and replaced with a medium containing 500 μg/mL *L. czeczottianus* and 1,000 μg/mL *L. nissolia* methanolic extracts, which are close to IC_50_ values. After 72 h, the cells were washed with 1X phosphate buffered saline (PBS) to remove cellular debris and photographed under an inverted microscope Leica DM IL LED using a DFC-290 camera (Leica, Wetzlar, Germany).

### Statistical analysis

For all the experiments, the assays were carried out in triplicate. The results are expressed as mean values and standard deviation (SD). The differences between the different extracts were analyzed using one-way analysis of variance (ANOVA) followed by Tukey's honestly significant difference *post-hoc* test with α = 0.05. This treatment was carried out using SPSS v. 14.0 program.

## Results and discussion

### HPLC-ESI-MS^n^

**Figure 1:**
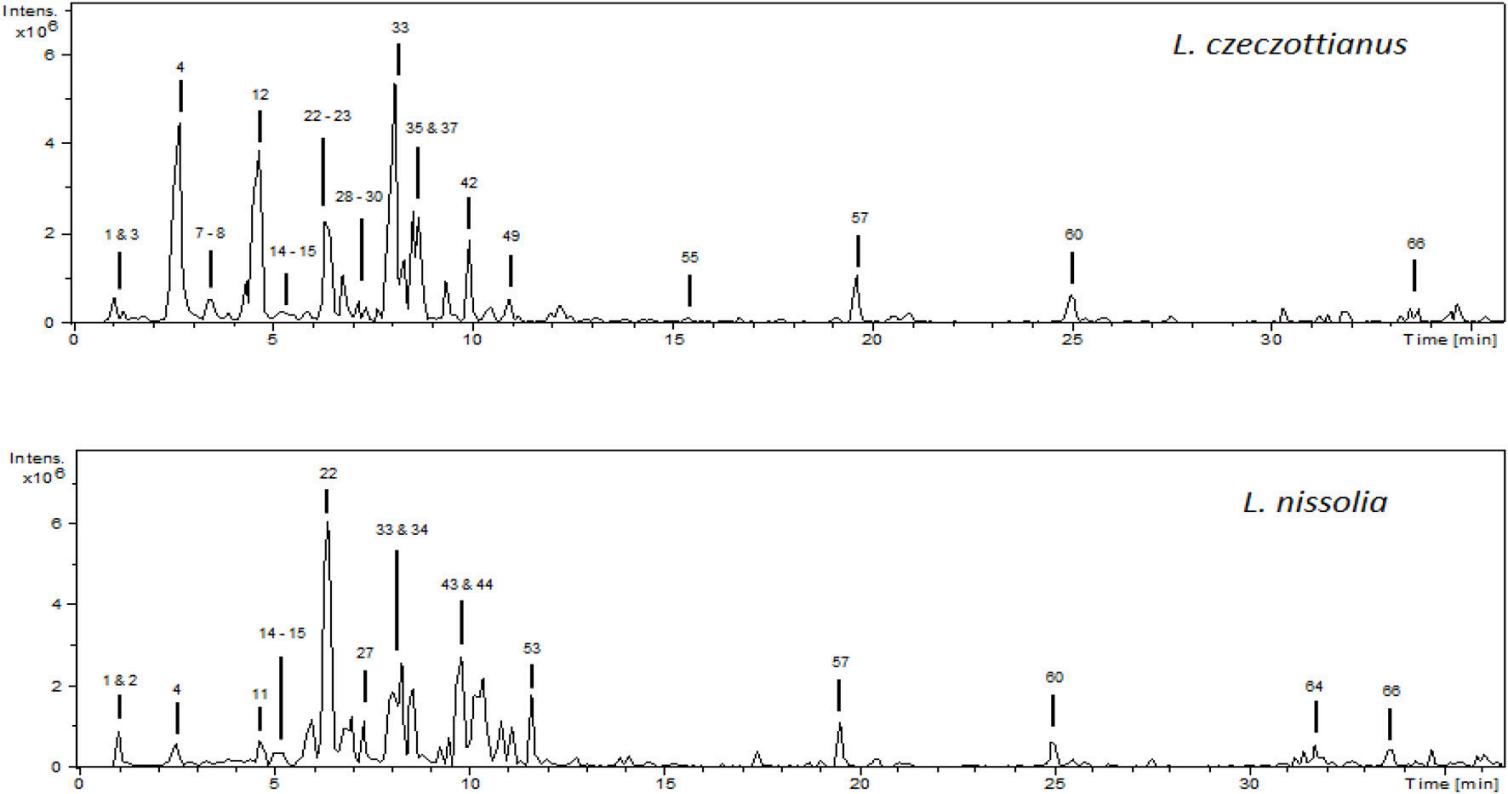
**HPLC-ESI/MS^n^ base peak chromatograms (BPC) of the methanolic extracts from *L. czeczottianus and L. nissolia***.

The analysis of the phenolic composition in aerial parts of *L. czeczottianus and L. nissolia* was performed by HPLC-ESI-MS^n^ using the negative ionization mode. Two independent assays were carried out for each sample, obtaining similar data regarding the nature and relative intensities of the detected fragments. The base peak chromatograms of the methanolic extracts are shown in Figure 1.

The initial step for the characterization of the phenolic compounds consisted in the determination of the molecular weight of each compound. In the negative ionization mode (ESI^−^) MS^1^ spectrum, the most intense peak usually corresponded to the deprotonated molecular ion [M-H]^−^. Compounds were numbered in both chromatograms by their order of elution. The tentative characterization of the detected compounds is shown in Table 1, and the discussion of the characterization is explained in the following sub-sections.

**Table 1:** **Characterization of the methanolic extract of aerial parts from *L. czeczottianus* (S1) and *L. nissolia* (S2)**.

**No**.	**t*_R_*(min)**	**[M-H]*m/z***	**m/z (% base peak)**	**Assigned identification**	**Sample**	**References**
1	1.1	191	MS^2^ [191]: 173 (41), 111 (100)	Citric acid	S1, S2	Flores et al., 2012
2	1.1	377	MS^2^ [377]: 341 (100), 215 (21) MS^3^ [377→341]: 179 (100), 161 (20), 143 (28), 119 (19), 113 (25)	Oligosaccharide derivative	S2	—
3	1.5	315	MS^2^ [315]: 153 (100) MS^3^ [315→153]: 109 (95), 108 (100)	Dihydroxybenzoic acid hexoside	S1	Engels et al., 2012
4	2.7	353	MS^2^ [353]: 191 (100), 179 (3), 173 (4)	5-O-caffeoylquinic acid	S1, S2	Clifford et al., 2003
5	2.9	325	MS^2^ [325]: 163 (100) MS^3^ [325→163]: 119 (100)	Coumaric acid-O-hexoside	S2	—
6	3.1	609	MS^2^ [609]: 519 (21), 489 (100), 447 (10), 369 (22) MS^3^ [609→489]: 471 (9), 399 (42), 369 (100)	Unknown	S1	—
7	3.4	865	MS^2^ [865]: 739 (88), 713 (26), 695 (100), 577 (65), 575 (35), 289 (29), 287 (30)	(Epi)catechin-(epi)catechin-(epi)catechin (B-type)	S1	Kajdžanoska et al., 2010
8	3.6	577	MS^2^ [577]: 451 (21), 425 (100), 407 (64), 289 (23), 287 (10), 245 (4)	(Epi)catechin-(epi)catechin (B-type)	S1	Kajdžanoska et al., 2010
9	3.9	755	MS^2^ [755]: 593 (100) MS^3^ [755→593]: 285 (100) MS^4^[755→593→285]: 257 (100), 229 (47)	Kaempferol-O-hexoside-O-rutinoside	S2	—
10	4.5	289	MS^2^ [289]: 245 (100), 205 (42), 203 (24), 179 (20)	Epicatechin	S1	Stöggl et al., 2004
11	4.6	785	MS^2^ [785]: 623 (100) MS^3^ [785→623]: 315 (100), 300 (16), 271 (6)	Isorhamnetin -O-hexoside-O-rutinoside	S2	—
12	4.7	593	MS^2^ [593]: 575 (10), 503 (29), 473 (100), 383 (38), 353 (99) MS^3^ [593→473]: 383 (16), 353 (100) MS^4^[593→473→353]: 325 (100), 297 (56)	Vicenin-2 (apigenin 6,8-di-C-hexoside)	S1	Llorent-Martínez et al., 2015
13	4.9	339	MS^2^ [339]: 191 (40), 179 (100), 173 (73), 161 (88), 135 (63) MS^3^ [339→179]: 135 (100)	Caffeic acid derivative	S1	—
14	5.3	563	MS^2^ [563]: 473 (100), 455 (45), 403 (26), 383 (88), 353 (85), 295 (44)	Apigenin-C-hexoside-C-pentoside	S1, S2	Han et al., 2008
15	5.3	579	MS^2^ [579]: 561 (11), 519 (9), 489 (73), 459 (100), 399 (42), 369 (43)	Luteolin-C-hexoside-C-pentoside	S1, S2	Algamdi et al., 2011
16	5.4	865	MS^2^ [865]: 739 (48), 713 (28), 695 (100), 577 (44), 575 (13), 407 (27), 289 (10), 287 (23)	(Epi)catechin-(epi)catechin-(epi)catechin (B-type)	S1	Kajdžanoska et al., 2010
17	5.7	367	MS^2^ [367]: 191 (100), 173 (13)	5-feruloylquinic acid	S1	Clifford et al., 2003
18	5.8	771	MS^2^ [771]: 609 (100) MS^3^ [771→609]: 301 (100), 300 (18) MS^4^[771→609→301]: 271 (56), 255 (41), 179 (100), 151 (80)	Quercetin-O-hexoside-O-rutinoside	S2	—
19	5.9	593	MS^2^ [593]: 593 (100), 473 (29), 431 (19), 311 (27)	Apigenin-C-hexoside-O-hexoside	S1	Zhang et al., 2011
20	6.1	625	MS^2^ [625]: 463 (12), 445 (44), 301 (50), 300 (100), 271 (45) MS^3^ [625→300]: 179 (100), 151 (41)	Quercetin-O-hexoside-O-hexoside	S1, S2	—
21	6.2	609	MS^2^ [609]: 447 (70), 285 (100) MS^3^ [609→285]: 151 (100)	Kaempferol-O-hexoside-O-hexoside	S2	—
22	6.5	563	MS^2^ [563]: 503 (42), 473 (100), 443 (98), 383 (71), 353 (54)	Apigenin-6-C-arabinoside-8-C-glucoside (isoschaftoside)	S1, S2	Ferreres et al., 2003
23	6.5	447	MS^2^ [447]: 429 (25), 357 (87), 327 (100) MS^3^ [447→357]: 339 (66), 297 (100), 285 (15) MS^3^ [447→327]: 309 (6), 299 (100), 285 (33)	Luteolin-6-C-hexoside (isoorientin)	S1, S2	Waridel et al., 2001
24	6.6	593	MS^2^ [593]: 473 (100), 447 (3), 429 (60), 357 (27), 339 (11), 327 (26), 309 (17) MS^3^ [593→473]: 327 (100), 298 (35)	2″-*O*-Rhamnosyl isoorientin	S2	Figueirinha et al., 2008
25	7.0	367	MS^2^ [367]: 191 (35), 179 (100), 135 (66)	Feruloylquinic acid	S1	—
26	7.1	593	MS^2^ [593]: 503 (16), 473 (100), 383 (55), 353 (43) MS^3^ [593→473]: 353 (100)	Apigenin-di-C-hexoside	S1	—
27	7.3	563	MS^2^ [563]: 503 (15), 473 (61), 443 (100), 383 (45), 353 (71)	Apigenin-C-hexoside-C-pentoside	S2	Han et al., 2008
28	7.4	739	MS^2^ [739]: 739 (100), 285 (8), 284 (7) MS^3^ [739→284]: 255 (100), 241 (22), 151 (59)	Kaempferol derivative	S1	—
29	7.4	609	MS^2^ [609]: 447 (13), 429 (39), 285 (100), 284 (54) MS^3^ [609→285]: 255 (100), 151 (82)	Kaempferol-O-hexoside-O-hexoside	S1	—
30	7.4	521	MS^2^ [521]: 359 (100) MS^3^ [521→359]: 344 (100)	Methylated flavonoid-O-hexoside	S1	—
31	7.7	755	MS^2^ [755]: 301 (100) MS^3^ [755→301]: 179 (31), 151 (100)	Quercetin derivative	S1	—
32	7.7	593	MS^2^ [593]: 447 (100) MS^3^ [593→447]: 301 (100), 300 (23) MS^4^[593→447→301]: 179 (100), 151 (30)	Quercetin-O-deoxyhexoside-O-deoxyhexoside	S2	—
33	8.1	431	MS^2^ [431]: 341 (13), 311 (100) MS^3^ [431→311]: 284 (25), 283 (100)	Vitexin	S1, S2	Gouveia and Castilho, 2011
34	8.1	609	MS^2^ [609]: 301 (100), 300 (20) MS^3^ [609→301]: 179 (100), 151 (27)	Rutin	S2	—
35	8.4	463	MS^2^ [463]: 301 (100), 300 (14) MS^3^ [463→301]: 271 (32), 255 (24), 179 (100), 151 (68)	Quercetin-O-hexoside	S1, S2	—
36	8.6	607	MS^2^ [607]: 443 (100), 323 (55) MS^3^ [607→443]: 323 (100), 308 (23)	Unknown	S2	—
37	8.7	739	MS^2^ [739]: 285 (100) MS^3^ [739→285]: 257 (100), 151 (89)	Kaempferol derivative	S1	—
38	9.0	477	MS^2^ [477]: 315 (100) MS^3^ [477→315]: 300 (100)	Isorhamnetin -O-hexoside	S2	—
39	9.3	609	MS^2^ [609]: 609 (100), 301 (28), 300 (38), 271 (12) MS^3^ [609→300]: 271 (100), 255 (89), 179 (21)	Quercetin-O-neohesperidoside	S2	—
40	9.5	433	MS^2^ [433]: 301 (77), 300 (100) MS^3^ [433→300]: 271 (90), 255 (64), 179 (100), 151 (18)	Quercetin-O-pentoside	S1	—
41	9.5	447	MS^2^ [447]: 284 (100), 255 (16) MS^3^ [447→284]: 255 (100), 227 (9)	Kaempferol-O-hexoside	S1	—
42	9.8	515	MS^2^ [515]: 353 (100) MS^3^ [515→353]: 191 (100), 179 (92), 173 (22), 135 (19)	3,5-dicaffeoylquinic acid	S1	Clifford et al., 2003
43	9.9	577	MS^2^ [577]: 269 (100) MS^3^ [577→269]: 225 (100)	Apigenin-O-rutinoside	S2	—
44	9.9	623	MS^2^ [623]: 315 (100), 300 (16), 271 (10)	Isorhamnetin -O-rutinoside	S2	—
45	10.2	607	MS^2^ [607]: 299 (100), 284 (17) MS^3^ [607→299]: 284 (100)	Methyl-flavonoid-O-rutinoside	S2	—
46	10.4	447	MS^2^ [447]: 285 (100)	Kaempferol/luteolin-O-hexoside	S2	—
47	10.5	417	MS^2^ [417]: 284 (100) MS^3^ [417→284]: 255 (100), 227 (5)	Kaempferol-O-pentoside	S1	—
48	10.6	431	MS^2^ [431]: 269 (100) MS^3^ [431→269]: 225 (100)	Apigenin-O-hexoside	S2	—
49	11.0	515	MS^2^ [515]: 353 (100), 173 (20) MS^3^ [515→353]: 191 (39), 179 (47), 173 (100)	4,5-dicaffeoylquinic acid	S1	Clifford et al., 2003
50	11.0	491	MS^2^ [491]: 329 (100) MS^3^ [491→329]: 314 (100), 299 (28)	Compound **56**-O-hexoside	S2	—
51	11.2	359	MS^2^ [359]: 197 (21), 179 (34), 161 (100), 133 (10)	Rosmarinic acid	S1	Liu et al., 2007
52	11.2	461	MS^2^ [461]: 299 (100) MS^3^ [461→299]: 284 (100), 151 (2)	Methyl-flavonoid-O-hexoside	S2	—
53	11.6	477	MS^2^ [477]: 315 (100), 300 (10) MS^3^ [477→315]: 300 (100)	Isorhamnetin-O-hexoside	S2	—
54	14.4	301	MS^2^ [301]: 179 (79), 151 (100)	Quercetin	S1	—
55	15.1	677	MS^2^ [677]: 515 (100), 353 (27), 335 (7) MS^3^ [677→515]: 353 (100), 335 (25), 191 (15), 173 (18)	Tricaffeoylquinic acid	S1	Han et al., 2008
56	18.7	329	MS^2^ [329]: 314 (100) MS^3^ [329→314]: 299 (100) MS^4^[329→314→299]: 271 (51), 255 (22), 227 (100), 161 (81)	Dimethyl-flavonoid	S2	—
57	19.5	327	MS^2^ [327]: 291 (32), 229 (76), 211 (44), 209 (13), 171 (100)	Oxo-dihydroxy-octadecenoic acid	S1, S2	Van Hoyweghen et al., 2014
58	20.5	327	MS^2^ [327]: 309 (13), 229 (56), 211 (26), 209 (12), 171 (100)	Oxo-dihydroxy-octadecenoic acid	S1, S2	Van Hoyweghen et al., 2014
59	20.9	391	MS^2^ [391]: 347 (100), 305 (35), 247 (39), 213 (38)	Unknown	S1	
60	25.0	329	MS^2^ [329]: 311 (12), 293 (23), 229 (100), 211 (77), 171 (42)	Trihydroxy-octadecenoic acid	S1, S2	Van Hoyweghen et al., 2014
61	25.4	359	MS^2^ [359]: 344 (100), 329 (22) MS^3^ [359→344]: 329 (100) MS^4^[359→344→329]: 314 (29), 311 (100), 283 (25)	Trimethyl-flavonoid	S2	—
62	26.9	971	MS^2^ [971]: 809 (100), 791 (3), 629 (9), 585 (9) MS^3^ [971→809]: 629 (100)	Hex-Hex-HexA-Hederagenin	S1	Pollier et al., 2011
63	30.2	941	MS^2^ [941]: 779 (100), 617 (19) MS^3^ [941→779]: 617 (100)	Unknown	S1	—
64	31.7	343	MS^2^ [343]: 328 (100), 313 (18) MS^3^ [343→328]: 313 (100) MS^4^[343→328→313]: 298 (100), 285 (343), 271 (13)	Trimethyl-flavonoid	S2	—
65	31.8	691	MS^2^ [691]: 415 (100), 397 (24), 293 (36) MS^3^ [691→415]: 253 (100), 179 (73), 143 (20), 131 (17)	Unknown	S1, S2	—
66	33.6	941	MS^2^ [941]: 923 (100), 879 (31), 795 (8), 751 (7), 733 (22), 615 (6), 525 (12) MS^3^ [941→923]: 879 (92), 733 (100), 597 (48)	3-Rha-Gal-GlcA-Soyasapogenol B	S1, S2	Pollier et al., 2011

#### Phenolic acids

Compound **3** exhibited the deprotonated molecular ion at m/z 315, and suffered the neutral loss of 162 Da (hexoside) to yield a fragment ion at m/z 153. It was characterized as dihydroxybenzoic acid hexoside (Engels et al., 2012).

Four caffeoylquinic acids were detected in the analyzed extracts. Compound **4**, with [M-H]^−^ at m/z 353, base peak ion at m/z 191, and low intensity at m/z 179, was characterized as 5-caffeoylquinic acid (Clifford et al., 2003). Compounds **42** and **49** exhibited the deprotonated molecular ions at m/z 515 and typical base peaks at m/z 353. Following the hierarchical key for the identification of di-caffeoylquinic acids (Clifford et al., 2003), **42** and **49** were identified as 3,5-dicaffeoylquinic acid and 4,5-dicaffeoylquinic acid, respectively. Finally, compound **55** was characterized as a tricaffeoylquinic acid due to its [M-H]^−^ ion at m/z 677 and MS^2^ and MS^3^ base peak ions at m/z 515 and 353(Han et al., 2008).

Compound **5**, with [M-H]^−^ at m/z 325, suffered the neutral loss of 162 Da, yielding a fragment ion at m/z 163 (coumaric acid, due to its typical fragment ion at m/z 119). Hence, it was identified as coumaric acid-O-hexoside.

Compound **10** was identified as epicatechin considering its mass spectrum and retention time.

Compound **13** exhibited the deprotonated molecular ion at m/z 339, and the 179→135 transition, typical from caffeic acid, was observed in the MS^3^ mass spectrum. Without further information, it was tentatively characterized as a caffeic acid derivative.

Compound **17**, with [M-H]^−^ at m/z 367, displayed the base peak ion at m/z 191 and a secondary fragment ion at m/z 173. According to (Clifford et al., 2003), it was identified as 5-feruloylquinic acid. Compound **25** was also characterized as a feruloylquinic acid.

Compound **51** exhibited the deprotonated molecular ion at m/z 359 and presented fragment ions at m/z 197, 179, 161, and 133, which are typical of rosmarinic acid (Liu et al., 2007).

#### Flavonoids

Most of the observed compounds in the analyzed extracts were flavonoids. They will be discussed based on their aglycone. The mass spectra of the glycosides showed the aglycone ions as a result of the loss of moieties such as hexosyl (162 Da), pentosyl (132 Da), or rutinosyl (308 Da).

Seven kaempferol derivatives were detected, all of them showing the aglycone kaempferol at m/z 285 (kaempferol standard was analyzed to compare its mass spectrum). Compound **9**, with [M-H]^−^ at m/z 755, suffered consecutive losses of 162 and 308 Da, and was tentatively characterized as kaempferol-O-hexoside-O-rutinoside. Compounds **21** and **29** exhibited the deprotonated molecular ion at m/z 609 and suffered two losses of 162 Da, so they were characterized as kaempferol-O-hexoside-O-hexoside. Compounds **28** and **37** were merely characterized as kaempferol derivatives as the only fragment ion observed in MS^2^ corresponded to kaempferol, and the identity of the attached moieties could not be elucidated. Compounds **41** and **47** exhibited neutral losses of hexoside and pentoside moieties, respectively, and were characterized as kaempferol-O-hexoside and kaempferol-O-pentoside, respectively.

Four compounds exhibited the aglycone isorhamnetin (identified by its 315→300 transition) in their fragmentation patterns. Compound **11**, with [M-H]^−^ at m/z 785, suffered consecutive neutral losses of 162 Da (hexoside) and 308 Da (rutinoside), yielding isorhamnetin at m/z 315, so it was characterized as isorhamnetin-O-hexoside-O-rutinoside. In a similar way, compounds **38** and **53** were identified as isorhamnetin-O-hexosides, and **44** as isorhamnetin-O-rutinoside.

Nine apigenin derivatives were tentatively characterized, most of them C-glycosides. Compound **12** was identified as vicenin-2 (apigenin-6,8-di-C-hexoside) considering bibliographic data (Llorent-Martínez et al., 2015). With a similar fragmentation pattern, compound **26** was also characterized as an apigenin-di-C-hexoside. The mass spectra of compounds **14**, **22**, and **27** was consistent with apigenin-C-hexoside-C-pentoside (Han et al., 2008). Due to its high abundance, compound **22** was selected for molecular docking simulations. According to scientific bibliography (Ferreres et al., 2003), the high relative intensity of the ion at m/z 503, [M-H-60]^−^, indicates that the pentose unit is in position 6. Hence, **22** was characterized as isoschaftoside (apigenin-6-C-arabinoside-8-C-glucoside). Compound **19**, with [M-H]^−^ at m/z 593, and fragment ions at m/z 473, 431, and 311, was tentatively characterized as apigenin-C-hexoside-O-hexoside (Zhang et al., 2011). Compound **33** was characterized as apigenin-8-C-glucoside (vitexin) according to Gouveia and Castilho (2011). Finally, compounds **43** and **48** exhibited neutral losses of 308 and 162 Da, respectively, to yield the aglycone apigenin at m/z 269 (characteristic fragment ion at m/z 225); therefore, they were identified as apigenin-O-rutinoside and apigenin-O-hexoside, respectively.

Three luteolin glycosides were detected. Compound **15** exhibited the deprotonated molecular ion at m/z 579, and the fragment ions at m/z 561, 519, 489, 459, 399, and 369 were consistent with luteolin-C-hexoside-C-pentoside (Algamdi et al., 2011). Compound **23** was tentatively characterized as isoorientin (luteolin-6-C-hexoside) after comparison of its fragmentation pattern with bibiliographic data (Waridel et al., 2001). Finally, compound **24** was characterized as 2″*-O-r*hamnosyl isoorientin (Figueirinha et al., 2008).

Two compounds presented the deprotonated molecular ion at m/z 609, and similar fragmentation patterns. Compound **34** was unambiguously identified as rutin after comparison with an analytical standard, whereas compound **39** was tentatively identified as quercetin-O-neohesperidoside.

Compound **54**, with [M-H]^−^ at m/z 301 and fragment ions at m/z 179 and 151, was identified as quercetin after comparison with an analytical standard. Six quercetin derivatives were detected in the analyzed extracts. The characterization of compounds **18**, **20**, **32**, **35**, and **40** (Table 1) was carried out considering the observed neutral losses of 162 Da (hexoside), 132 Da (pentoside), 146 Da (deoxyhexoside) and 308 Da (rutinoside), to yield the aglycone quercetin at m/z 301. Compound **31** was merely characterized as a quercetin derivative.

Several methylated flavonoids, as aglycones or as glycosides, were tentatively characterized. Compound **56**, with [M-H]^−^ at m/z 329, suffered two consecutive losses of methyl groups (15 Da), and was characterized as a dimethyl-flavonoid. Compound **50** suffered the neutral loss of 162 Da to yield the aglycone, which exhibited the same fragmentation pattern than **56**, so it was characterized as its O-hexoside. Compounds **30** and **52** suffered neutral losses of 162 Da, yielding aglycones that presented the pattern of methylated flavonoids, so they were characterized as methyl-flavonoid-O-hexoside. In a similar way, **45** was assigned to a methyl-flavonoid-O-rutinoside. Finally, compounds **61** and **64** suffered three losses of methyl groups and were characterized as trimethyl-flavonoids.

#### Other compounds

Compound **1**, with the deprotonated molecular ion at m/z 191 and MS^2^ base peak at m/z 111, was identified as citric acid (Flores et al., 2012).

Compound **2** presented MS^n^ fragment ions at m/z 341, 179, 161, 143, 119, and 113, typical of hexoses (Brudzynski and Miotto, 2011), and was tentatively characterized as an oligosaccharide derivative.

Three B-type proanthocyanidins were detected in the extracts of *L. czeczottiaus*. Compounds **7** and **16**, with [M-H]^−^ at m/z 865, were characterized as procyanidin trimers of B-type considering their MS^2^ fragment ions and consecutive losses of 288 Da, previously reported in bibliography for (epi)catechin-(epi)catechin-(epi)catechin (Kajdžanoska et al., 2010). Compound **8** exhibited its base peak ions at m/z 577, and fragment ions at m/z 451, 425, 407, 289, and 287, typical from procyanidin dimers of the type (epi)catechin-(epi)catechin (Ruiz et al., 2005; Kajdžanoska et al., 2010).

By comparison of their fragmentation patterns and bibliographic information (Van Hoyweghen et al., 2014), **57** and **58** were characterized as oxo-dihydroxy-octadecenoic acids, and **60** as trihydroxy-octadecenoic acid.

Finally, two saponins were characterized in the analyzed extracts. Compounds **62** and **66**, with deprotonated molecular ions at m/z 971 and 941, respectively, were identified after comparison of their fragmentation patterns (Table 1) with bibliographic data (Pollier et al., 2011).

### Antioxidant properties

Phenolic compounds are known to serve as multifunctional bioactive components (anti-oxidant, anti-microbial, anti-inflammatory and anti-cancer agents). Several studies also concluded that these components are main contributors on the antioxidant effects of plant extracts (Shahidi and Ambigaipalan, 2015). In this context, they were selected as target compounds in the present study. Total phenolic and flavonoid contents were detected by colorimetric methods, namely Folin-Ciocβlteu and AlCl_3_. The results are shown in Table 2. The total phenolic content in *L. czeczottianus* (63.16 mgGAE/g extract) was higher than in *L. nissolia* (26.47 mgRE/g extract). However, *L. nissolia* (20.97 mgRE/g extract) had higher concentrations of flavonoids than *L. czeczottianus* (14.16 mgRE/g extract). Apparently, the total flavonoids accounted for more than 50% of the total phenolics in *L. nissolia* extract. From these results, flavonoids were thus the major components in *L. nissolia* extract. This finding was confirmed also by HPLC-ESI-MS^n^ analysis.

**Table 2:** **Extraction yields, total phenolic and flavonoid contents**.

**Species**	**Extraction yields (%)**	**Total phenolic content (mg GAE/g extract)**	**Total flavonoid content (mg RE/g extract)**
*L. czeczottianus*	7.93	63.16 ± 2.41^*^	14.16 ± 0.40
*L. nissolia*	14.94	25.47 ± 0.23	20.94 ± 0.78

* *Values expressed are means ±S.D. of three parallel measurements. GAE, Gallic acid equivalent; RE, Rutin equivalent*.

Free radicals trigger a wide variety of chronic and degenerative diseases, such as Alzheimer's disease, diabetes mellitus and cancer. At this point, DPPH and ABTS radicals are widely used to assess free radical scavenging ability of plant extracts or synthetic compounds. In these assays, the initial absorbances (blue for ABTS and purple for DPPH) are decreased by antioxidants, and these changes are spectrophotometrically measured (517 nm for DPPH and 734 nm for ABTS). As can be seen in Table 3, *L. czeczottianus* was found to exhibit potent free radical scavenging activities in both DPPH (IC_50_: 1.42 mg/mL) and ABTS (IC_50_: 1.80 mg/mL) assays. The observed remarkable free radical scavenging activity may be explained by the high level of phenolics in the extracts. This is in accordance with the data published by other authors, indicating that the higher the total phenolic contents, the better the free radical scavenging activity (Bannour et al., 2016; Zhang et al., 2016). Also, some phenolics were reported as effective hydrogen donors by means of their hydroxyl groups (Sevgi et al., 2015).

**Table 3:** **Antioxidant properties [IC_50_ and EC_50_ (in CUPRAC, FRAP phosphomolybdenum assays) values, mg/mL]**.

**Species**	**DPPH radical scavenging**	**ABTS radical scavenging**	**CUPRAC**	**FRAP**	**Phosphomolybdenum**	**Metal chelating**
*L. czeczottianus*	1.42 ± 0.04^*^	1.80 ± 0.01	0.56 ± 0.09	0.51 ± 0.04	1.29 ± 0.03	5>^**^
*L. nissolia*	3.19 ± 0.11	5>^**^	1.11 ± 0.01	1.38 ± 0.11	0.86 ± 0.05	1.14 ± 0.01
Trolox	0.07 ± 0.01	0.24 ± 0.01	0.20 ± 0.01	0.21 ± 0.01	0.41 ± 0.01	nt
EDTA	nt	nt	nt	nt	nt	0.02 ± 0.001

* *Values expressed are means ±S.D. of three parallel measurements. nt: not tested*.

** *The IC_50_ values were higher than 5 mg/mL*.

Reducing power is considered as an important antioxidant feature and it is reflected by the electron-donating ability of antioxidants. For this purpose, CUPRAC (Cu^2+^ →Cu^+^) and FRAP (Fe^3+^ →Fe^2+^) assays were performed. *L. czeczottianus* exhibited stronger reducing abilities in both assays as similar to the free radical scavenging abilities (Table 3). From these results, the high contents of phenolics in the extracts indicated that these compounds contribute to the higher reducing abilities. Similar results were obtained by several researchers, who reported a strong relationship between total phenolic content and reducing abilities (Beghlal et al., 2016; Smeriglio et al., 2016). Phosphomolybdenum assay is based on the reduction of Mo (VI) to Mo (V) by antioxidants at acidic pH; then, green phosphate-Mo (V) complex is formed, which has its maximum absorbance at 695 nm. The phosphomolybdenum activity was observed as lower for *L. czeczottianus* (EC_50_: 1.29 mg/mL) than *L. nissolia* (EC_50_: 0.86 mg/mL). (Table 3). Because phosphomolybdenum assay is considered as a total antioxidant capacity assay, our findings for this assay might be attributed also to non-phenolic antioxidants such as tocopherol or vitamin C. These results were consistent with other reports (Albayrak et al., 2010; Zengin et al., 2015). Also, some researchers observed a weak correlation between total phenolic content and phosphomolybdenum assay (Nićiforović et al., 2010; Sarikurkcu et al., 2015).

Transition metals (iron and copper) play an important role in Fenton and Haber-Weiss reactions, which are main reactions involved in the production of free radicals. In this sense, the chelating ability of these metals is considered to have a significant role among the antioxidant mechanisms. The metal chelating ability of *Lathyrus* extracts was determined by ferrozine method and tabulated in Table 3. In contrast to the free radical and reducing power assays, *L. nissolia* (IC_50_: 1.14 mg/mL) exhibited stronger metal chelating activity compared to *L. czeczottianus* (IC_50_: >5 mg/mL). Thus, the observed activity for *L. nissolia* may be caused from non-phenolic chelators, including peptides, polysaccharides or citric acid. In accordance with our results, some authors reported that the metal chelating activity of phenolics plays a minor role among antioxidant mechanisms, and is suggested by several researchers as a secondary antioxidant mechanism (Rice-Evans et al., 1996; Gursoy et al., 2009; Wang et al., 2009). To the best of our knowledge, this study is the first report on the antioxidant properties of these *Lathyrus* species. In this direction, the obtained results could be useful to provide new perspectives on the *Lathyrus* genus, and its species could be regarded as potential candidates for developing novel phyto-pharmaceuticals.

### Enzyme inhibitory properties

Alzheimer's disease (AD) and diabetes mellitus (DM) are major health problems due to the severe increase of their prevalence. For example, recent reports indicated DM prevalence to be 9% among adults, and it is supposed to be well above 15% until 2025 (Waltenberger et al., 2016). Today, more than 50 million people are also affected by AD, and this number is estimated to increase to 131.5 million people by 2,050 (Prince, 2015). Therefore, new effective strategies have vital importance in the treatment of these diseases (Rescigno et al., 2002; Mao et al., 2015; Zengin et al., 2016). Moreover, key enzyme inhibition theory is accepted as one of the most efficient strategies to counteract these diseases (Etxeberria et al., 2012; Murray et al., 2013; Mocan et al., 2016a,b). At this point, enzyme inhibitory assays are considered a very useful tool for investigating the biological properties of herbal extracts or isolated compounds (Bahadori et al., 2016; Dinparast et al., 2016). Considering the mentioned aspects, the enzyme inhibitory properties of *L. czeczottianus* and *L. nissolia* toward cholinesterases, α-amylase, α-glucosidase, and tyrosinase were investigated, and the results are gathered in Table 4.

**Table 4:** **Enzyme inhibitory properties (IC_50_ mg/mL)**.

**Species**	**AChE inhibiton**	**BChE inhibition**	**Tyrosinase inhibition**	**α- Amylase inhibition**	**α- Glucosidase inhibition**
*L. czeczottianus*	1.23 ± 0.02^*^	na	2.32 ± 0.10	3.87 ± 0.14	3.54 ± 0.27
*L. nissolia*	1.22 ± 0.05	5>^**^	na	3.66 ± 0.06	1.01 ± 0.01
Galantamine	0.003 ± 0.001	0.003 ± 0.001	nt	nt	nt
Kojic acid	nt	nt	0.13 ± 0.01	nt	nt
Acarbose	nt	nt	nt	1.27 ± 0.04	2.19 ± 0.10

* *Values expressed are means ±S.D. of three parallel measurements. na: not active; nt: not tested*.

** *The IC_50_ value was higher than 5 mg/mL*.

The inhibitory effects on cholinesterases of both species are modest, a higher effect being observed for *L. nissolia* (IC_50_: 1.22 mg/mL for AChE). Additionally, *L. czeczottianus* extract exhibited a high anti-tyrosinase effect, whereas *L. nissolia* was not active toward this enzyme. High anti-tyrosinase effects and modest inhibitory effects on cholinesterases were already reported in our previous studies concerning *L. aureus* and *L. pratensis* (Llorent-Martínez et al., 2016). Moreover, higher inhibitory effects toward α-glucosidase in comparison with α-amylase were observed in this study, the results being in line with our previous findings (Llorent-Martínez et al., 2016; Mocan et al., 2016b). Nonetheless, previous reports indicated that phenolic compounds have lower α-amylase inhibitory activity and a stronger inhibition activity against yeast α-glucosidase (Apostolidis et al., 2011). According to our results, the *Lathyrus* species have similar α-amylase inhibition and but *L. nissolia* exerted stronger α-glucosidase inhibition as compared to *L. czeczottianus*. Also, additional computational studies (molecular docking) were performed to understand possible interactions between the target compounds [5-O-CQA (**4**), isoschaftoside (**22**) and vitexin (**33**)] and tested enzymes as well as to get further insights into the modulation of biological response.

### Molecular modeling

**Figure 2:**
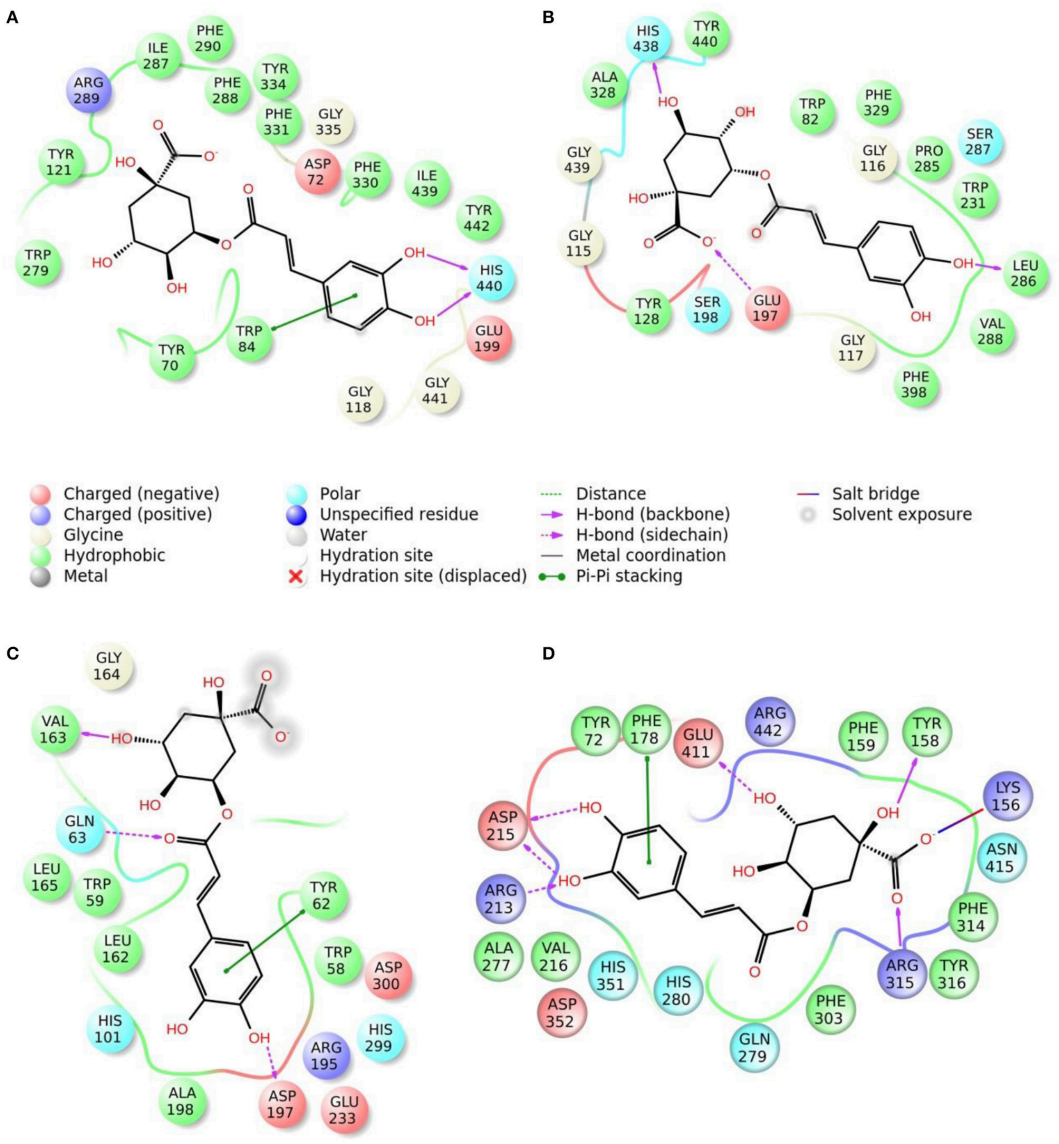
**Schematic representation of the interactions between the best pose found for 5-O-caffeoylquinic acid with (A)** AchE, **(B)** BchE, **(C)** α-amylase, **(D)** α-glucosidase.

**Figure 3:**
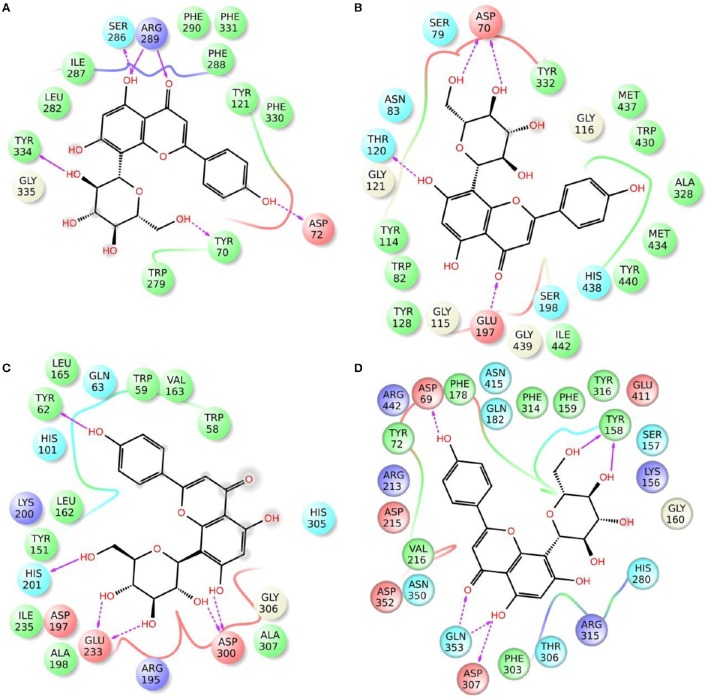
**Schematic representation of the interactions between the best pose found for vitexin with (A)** AchE, **(B)** BchE, **(C)** α-amylase, **(D)** α-glucosidase.

**Figure 4:**
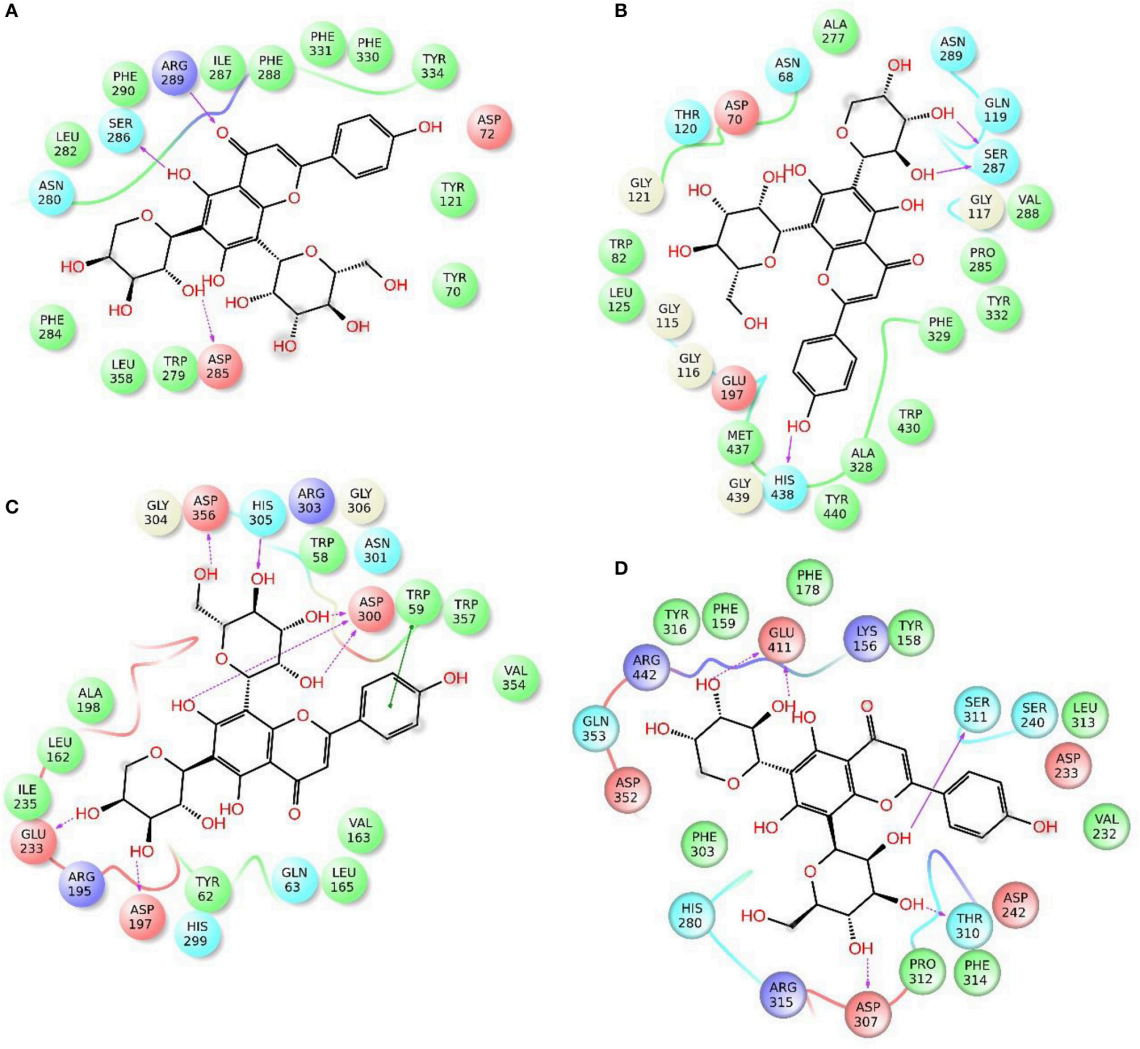
**Schematic representation of the interactions between the best pose found for isoschaftoside with (A)** AchE, **(B)** BchE, **(C)** α-amylase, **(D)** α-glucosidase.

**Figure 5:**
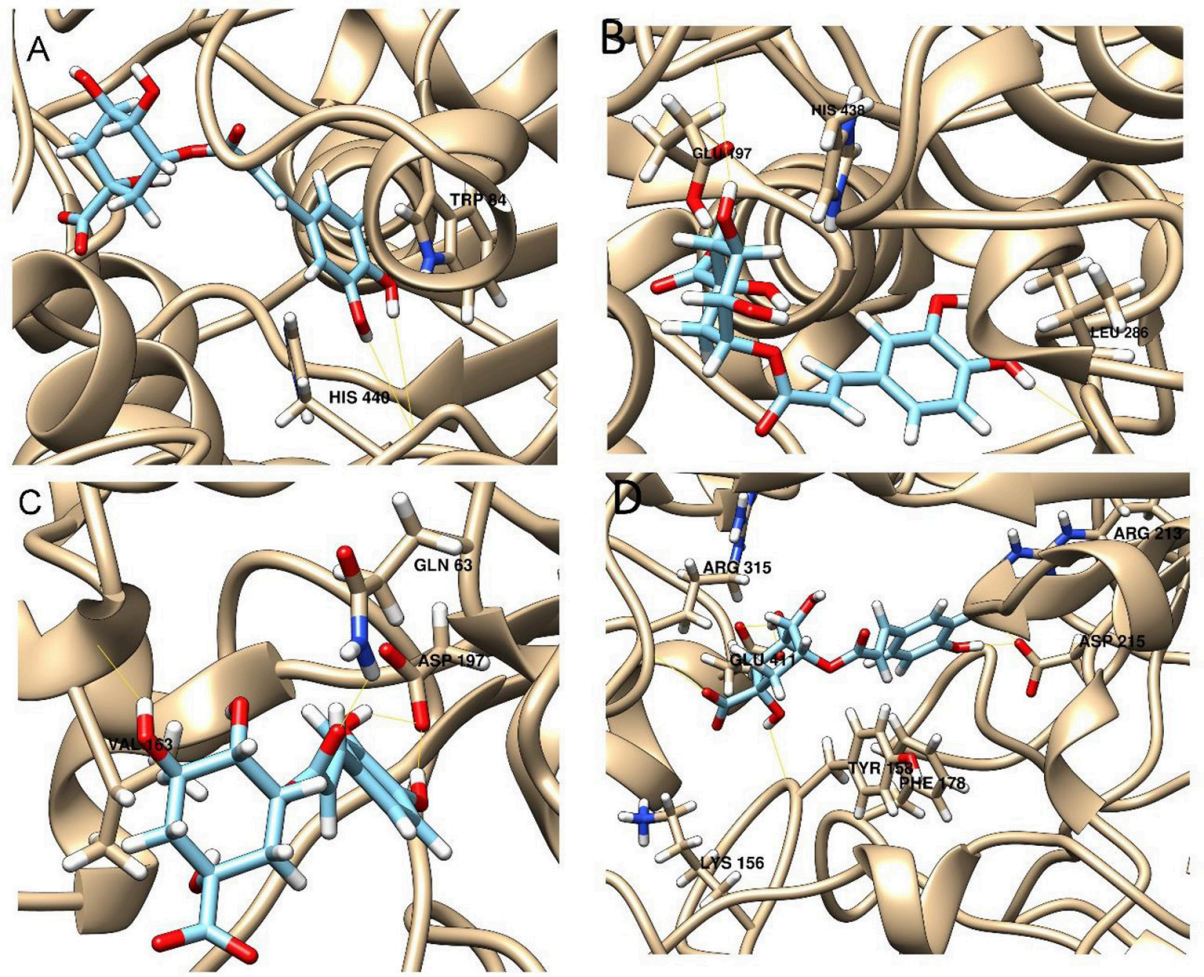
**Best pose of 5-O-caffeoylquinic acid with (A)** AchE, **(B)** BchE, **(C)** α-amylase, **(D)** α-glucosidase.

**Figure 6:**
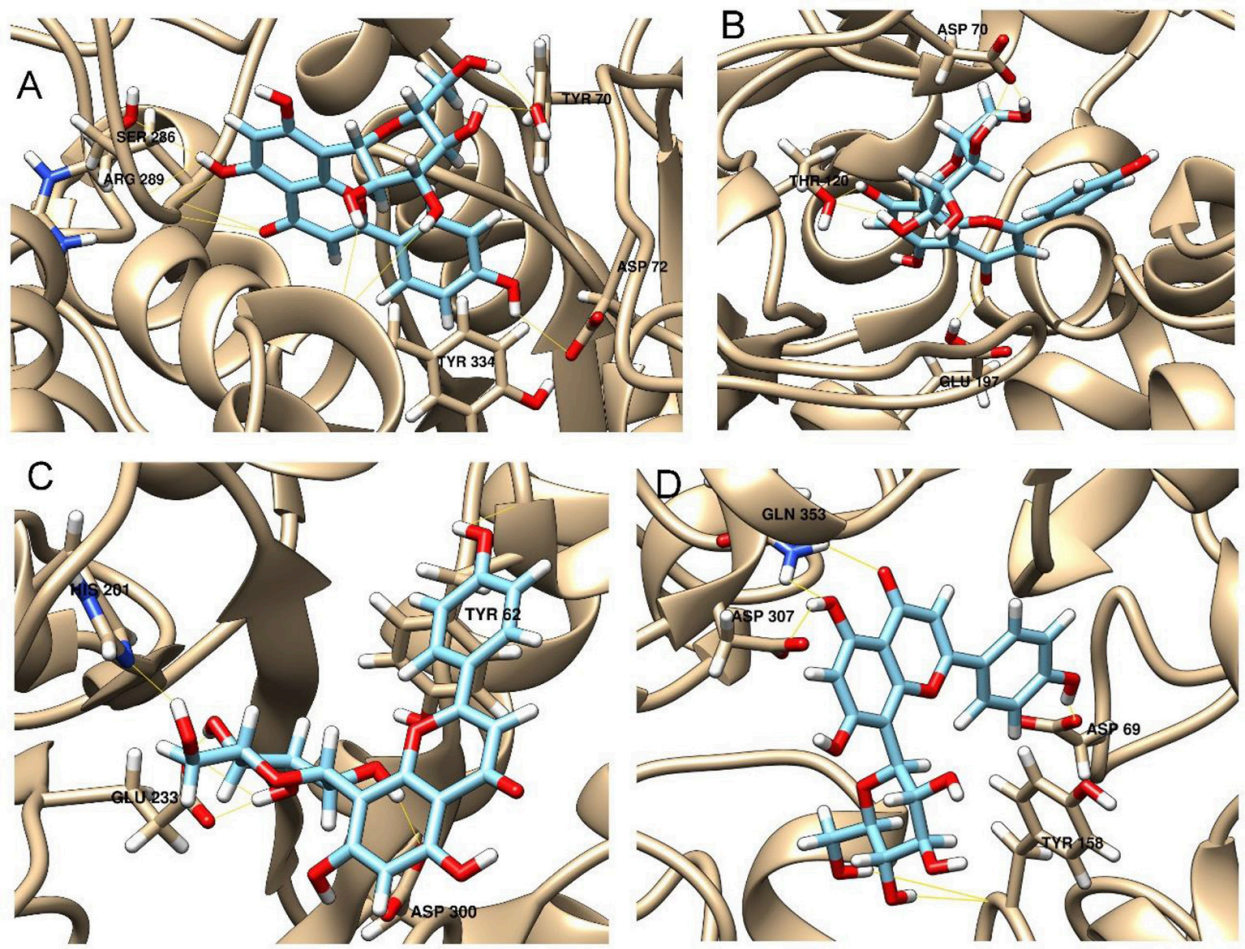
**Best pose of vitexin with (A)** AchE, **(B)** BchE, **(C)** α-amylase, **(D)** α-glucosidase.

**Figure 7:**
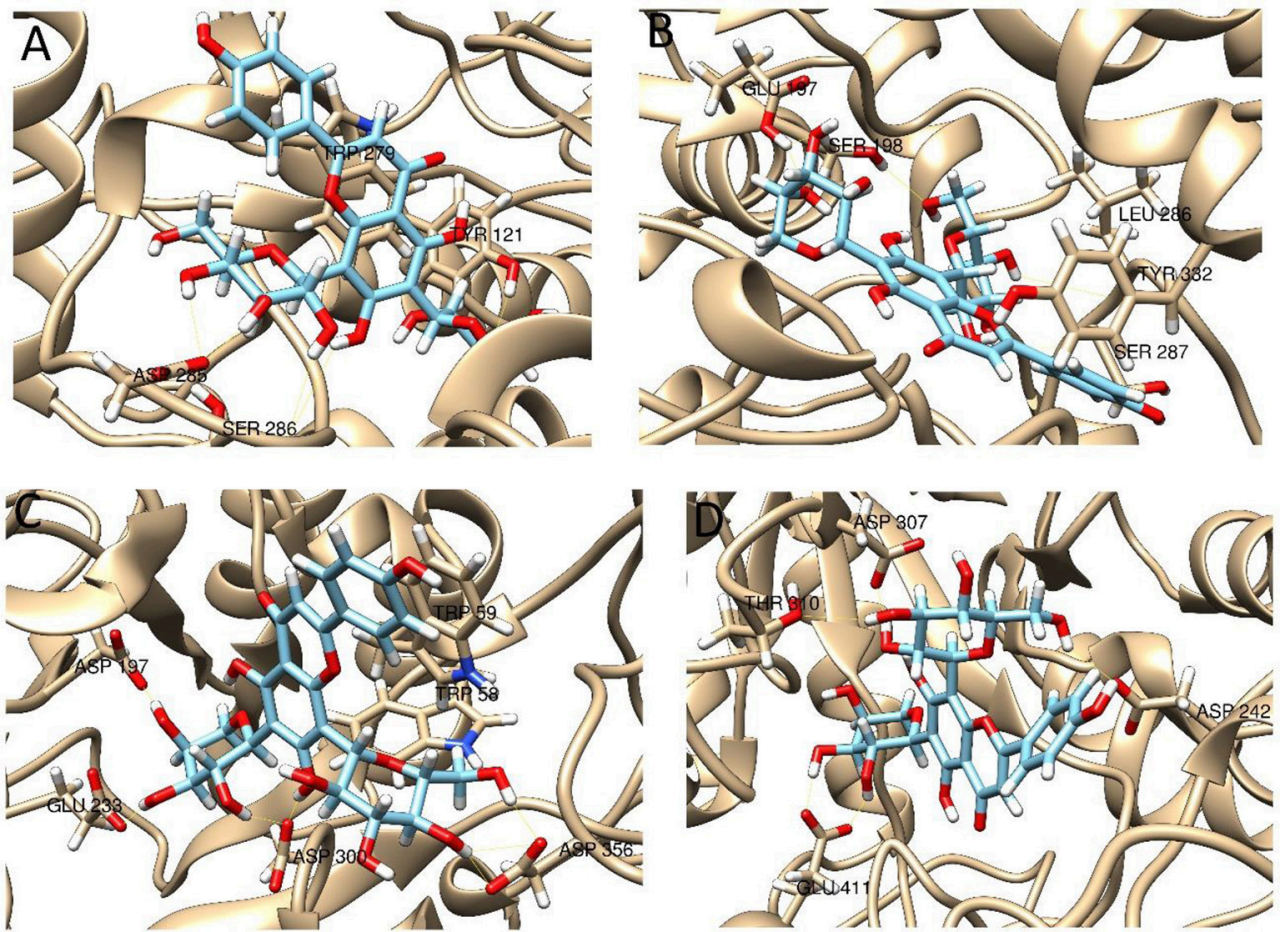
**Best pose of isoschaftoside with (A)** AchE, **(B)** BchE, **(C)** α-amylase, **(D)** α-glucosidase.

**Figure 8:**
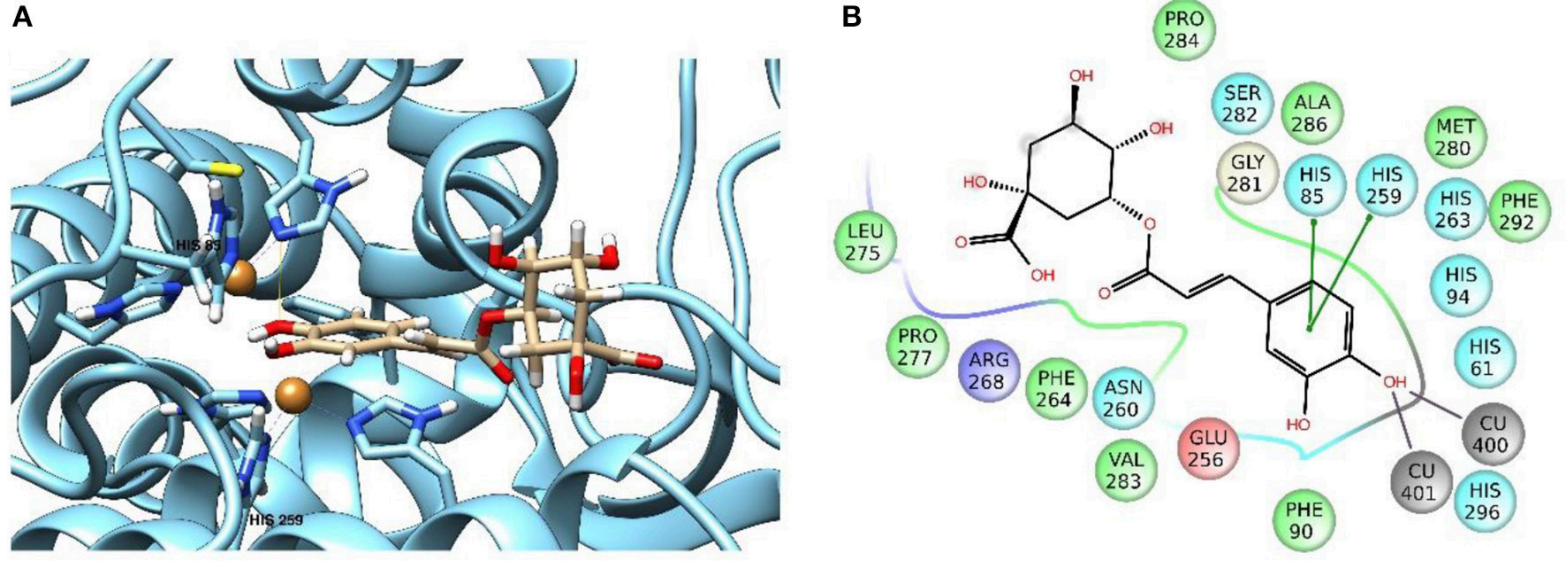
**Best pose of 5-O-caffeoylquinic acid (A)** and schematic representation of the interactions between the best pose found for 5-O-caffeoylquinic acid **(B)** to tyrosinase.

Enzymatic assays performed on the extract of *L. czeczottianus* evidenced a modest inhibition activity to AchE, and strong tyrosinase, whereas the *L. nissolia* extracts have shown relevant inhibition activity for AchE and α-glucosidase. The best and most representative enzyme-ligand complexes are reported in Figures 2–8. Among the identified bioactive compounds, vitexin (**33**) and 5-O-CQA (**4**), which were found to be dominant in both extracts (especially vitexin), and isoschaftoside (**22**) which is prevalent in the extract of *L. nissolia*, were selected for docking experiments. These compounds have been docked to AchE, BChE, α-glucosidase, α-amylase and tyrosinase.

The best pose found for 5-O-CQA (**4**) docked to AchE (docking score −9.01 Kcal/mol) was stabilized by several interactions with the residues surrounding the enzymatic pocket involving Trp84 with one π–π stack and His440 with two hydrogen bonds. The best ranked pose of vitexin (**33**) to the same enzyme (score = −8.30 Kcal/mol) interacts with the binding pocket of the AchE by two hydrogen bonds with Arg289, and one hydrogen bond with Ser286, Tyr334, Asp72, and Tyr70. The best pose found for iscoschaftoside (**22**) docked to AchE was stabilized by two hydrogen bonds to Ser286, two hydrogen bonds to Asp285, one hydrogen bond to Tyr121 and a π–π stack to Trp279 (score = −11.05 Kcal/mol). The best pose found for 5-O-CQA (**4**) in the enzymatic pocket of BchE was stabilized by the formation of one hydrogen bond with His438, Gln197 and Leu286 with a docking score = −9.39 Kcal/mol. The best pose for isoschaftoside (**22**) was stabilized by the formation of hydrogen bond with Glu197, Ser198, Leu286, Ser 287 and a π–π stack with Tyr332 (score = −10.08). The best pose found for vitexin (**33**) in the interaction to the enzymatic pocket of BchE was stabilized by the formation of three hydrogen bonds, respectively, with Gln197, Asp70, Thr120, and a much lower docking score: −11.24 Kcal/mol. These data are in agreement with the literature data in which isoschaftoside (**22**) has shown a high inhibitory activity to AchE and BChE with IC_50_ of 0.23 μM and 3.11 μM respectively (Hung et al., 2015).

5-O-CQA (**4**) binds to the enzymatic pocket of the α-amylase by forming a series of hydrogen bonds with Gln63, Val163, Asp197 residues (docking score = −8.50 Kcal/mol), whereas vitexin (**33**) interacted with the enzymatic pocket by establishing hydrogen bonds with Tyr62, His201, Glu233, and Asp300 (docking score = −8.27 Kcal/mol). Isoschaftoside (**22**) best pose established several hydrogen bonds to Asp356, Asp330, Glu233, Asp197, and Trp59, and also two π–π stacks to Trp58 and Trp59. Among the three tested substances, it presented the most favorable docking score (score = −9.69 Kcal/mol) to this enzyme. Several papers were found in scientific literature reporting the antidiabetic properties of some medicinal plants containing isoschaftoside (Song et al., 2012; Khazneh et al., 2016), so supporting the hypothesis that isoschaftoside may be a good inhibitor of α-amylase. In a previous study, vitexin (**33**) proved to be a potent α-glucosidase inhibitor with a reported EC50 = 0.4 mg/mL, close to that of Acarbose (Yao et al., 2011) and it was found that its best pose interacted with the enzymatic pocket of α-glucosidase by forming three hydrogen bonds with Arg315, Asp215, Arg213, and a π–π stack with the aromatic ring of Phe178 (docking score = −7.29 Kcal/mol). 5-O-CQA (**4**) interacted only by forming hydrogen bonds with Asp69, Trp158, Glu343, and Asp307 (Docking score = −8.83 Kcal/mol); isoschaftoside (**22**) interact by forming hydrogen bonds to Glu411, Asp307, Thr310, Asp242 (score = −7.73 Kcal/mol). For tyrosinase, the docking of vitexin (**33**), isoschaftoside (**22**) and 5-O-CQA (**4**) were significantly different. Vitexin (**33**) and isoschaftoside (**22**) were both unable to bind directly to the copper atoms present in the enzymatic pocket of tyrosinase also the interactions to the enzymatic pocket are marginal (see Figure 1S in the supporting material); on the contrary, the best pose found for 5-O-CQA (**4**) evidenced the ability of the molecule to coordinate one Cu atom present at the binding pocket of the enzyme, as depicted in Figure 8. This finding is in full agreement with our enzymatic assays, and also with the reported inhibitory activity found for some caffeoylquininic acid derivatives toward the tyrosinase (Iwai et al., 2004). In conclusion, the docking experiments performed in this work can explain the literature data regarding the inhibitory activity of vitexin (**33**), isoshaftoside (**22**) and 5-O-QCA (**4**), being capable to interact with the residues present in the binding pocket of the considered enzymes. In particular, vitexin (**33**), which has been reported to be efficacious to control blood glucose in diabetic rats (Choo et al., 2012), is present in the extracts of both plants and can be addressed as one of the compounds responsible for the α-glucosidase inhibitory activity found for both extracts together with isoschaftoside (**22**) which has shown a remarkable docking score on alpha-amylase, whereas 5-O-CQA (**4**), as demonstrated in the docking experiments, may efficaciously inhibit the tyrosinase activity by binding to one Cu atom present in the enzymatic pocket.

### Cytotoxicity assay

**Figure 9:**
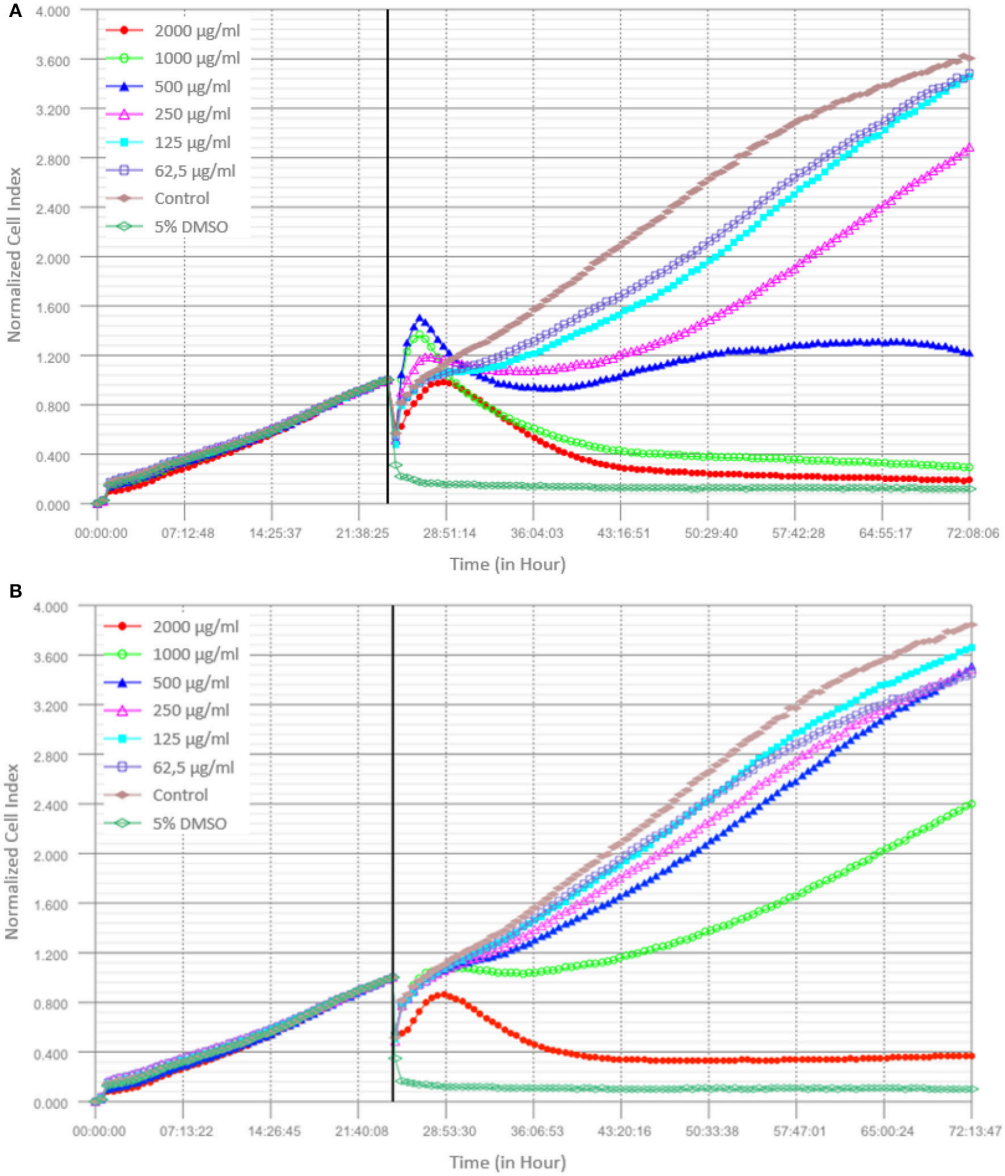
**Dynamic monitoring of the effects of different doses of *L. czeczottianus* (A)** and *L. nissolia* **(B)** extracts on HEK-293 cells by using iCELLigence real time cell analysis system.

**Figure 10:**
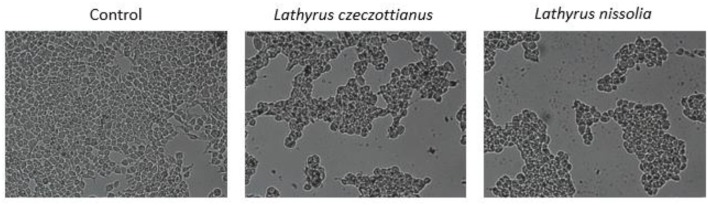
**Effects of *L. czeczottianus* and *L. nissolia* extracts on HEK-293 cell morphology and viability**. **Left panel**: Control cells. **Middle panel**: Cells were treated with 500 μg/mL *L. czeczottianus* extract. **Right panel**: Cells were treated with 1000 μg/mL *L. nissolia* extract.

Toxicity is a major issue to consider in drug screening or drug development from natural product derivatives. Some of the active compounds obtained from these natural products have biomedical uses such as anti-neoplastic and anti-inflammatory agents, and the toxicity of these compounds needs to be clarified by cytotoxicity assays in biological systems. In this study, we have tested the cytotoxicity of two different *Lathyrus* species (*L. czeczottianus* and *L. nissolia)* on human embryonic kidney cells, HEK-293, since these cells are widely used in drug toxicity studies as kidney may be one of the major target organ of several toxins (Mantle et al., 2015; Sang et al., 2016). Previous studies reported the use of HEK-293 cell line for neurobiological research as well, based on the findings that HEK-293 cells have many properties in common with neuronal precursors(Shaw et al., 2002; Thomas and Smart, 2005; Lin et al., 2014). For monitoring cytotoxicity, we used iCELLigence system, which enables to get real-time, label-free and non-invasive measurements from living cells (Bird and Kirstein, 2009; Martinez-Serra et al., 2014). In recent years, this system has been one of the gold standards in cell biology research since it measures the effect of the drugs without any additional treatment on the cells. In addition to this, RTCA systems have own data analysis software which calculates IC_50_ values automatically. In this study, IC_50_ values of *L. czeczottianus* and *L. nissolia* were determined as 413.19 ± 16.3 μg/mL and 1040.83 ± 48.8 μg/mL, respectively, from three independent experiments in a time and dose dependent manner after treating cells with six different concentrations of these two extracts (Figure 9). Furthermore, very high doses were tested, close to IC_50_ concentrations of the extracts, on HEK-293 cell morphology and almost 50% cellular viability were observed as seen in middle and right panels compared to control cells on the left (Figure 10). From these results, the studied extracts had no cytotoxic activities against HEK-293 cell lines. However, *L. czeczottianus* had higher cytotoxic effect with lower value of IC_50_ as compared to *L. nissolia*. In this direction, the observed remarkable activity for *L. nissolia* may be explained by the higher concentration of flavonoids in the extract. In fact, several authors reported that some flavonoids exhibited no cytotoxic effects on various cell lines (Kumar and Pandey, 2013; Sak, 2014). To the extent of our knowledge, only one previous study has been conducted on the cytotoxic evaluation of the *Lathyrus* species (L. *laxiflorus*) (Spanou et al., 2010).

## Conclusions

*Lathyrus* species are still an underestimated source of bioactive compounds, especially flavonoids, being poorly investigated up-to-date. The present study is the first report on the biological properties (antioxidant, enzyme inhibitory and cytotoxic effects) and phytochemical profiles of selected *Lathyrus* species. On one hand, *L. czeczottianus* exhibited remarkable antioxidant abilities, presenting the highest concentration of phenolics. On the other hand, *L. nissolia* had promising inhibitory effects on the studied enzymes, except for tyrosinase. Additionally, the extracts exhibited no cytotoxic effects on HEK-293 cell lines. Molecular docking experiments provided new insights on some major components (vitexin, isoschaftoside, and 5-O-caffeoylquininic acid) by studying their interactions with the enzyme pool, which gave the molecular basis of the biological response found for these species. According to our results, further studies on *Lathyrus* species could open new perspectives for developing novel health-promoting agents by pharmaceutical and nutraceutical industries.

## Author contributions

EJLM, GZ, MLFC, OB, ArA, RC, AnM, AdM, and SU set up and carried out experiments. GOG and AbA executed data analysis.

### Conflict of interest statement

The authors declare that the research was conducted in the absence of any commercial or financial relationships that could be construed as a potential conflict of interest.
