# Supplementary material for: Traditionally Used Lathyrus Species: Phytochemical Composition, Antioxidant Activity, Enzyme Inhibitory Properties, Cytotoxic Effects, and in silico Studies of L. czeczottianus and L. nissolia
Source: Front Pharmacol. 2017 Feb 27;8:83. doi: 10.3389/fphar.2017.00083 (PMC5326780; doi:10.3389/fphar.2017.00083)
Supplement: Supplementary file 1 [file DataSheet1.DOCX]

**Traditionally used *Lathyrus* species: Phytochemical composition, antioxidant activity, enzyme inhibitory properties, cytotoxic effects and *in silico* studies of *L. czeczottianus* and *L. nissolia***

Eulogio J. Llorent-Martínez ^1^, Gokhan Zengin^2*^, Maria Luisa Fernández-de Cordova^3^, Onur Bender^4^, Arzu Atalay^4^, Ramazan Ceylan^2^, Adriano Mollica^5^, Andrei Mocan^6*^, Sengul Uysal^2^, Gokalp Ozmen Guler^7^, Abdurrahman Aktumsek^2^

^1^ University of Castilla-La Mancha, Regional Institute for Applied Chemistry Research (IRICA), Ciudad Real 13071, Spain

^2^Selcuk University, Science Faculty, Department of Biology, Campus, 42250, Konya, Turkey

^3^Department of Physical and Analytical Chemistry, University of Jaén, Campus Las Lagunillas S/N, E-23071 Jaén, Spain

^4^Biotechnology Institute, Ankara University, 06100, Ankara, Turkey

^5^Department of Pharmacy, University “G. d’Annunzio” of Chieti-Pescara, 66100, Chieti-Italy

^6^Department of Pharmaceutical Botany, “Iuliu Hatieganu” University of Medicine and Pharmacy, 8, V. Babes Street, Cluj-Napoca, Romania

^7^Necmettin Erbakan University, Ahmet Kelesoglu Education Faculty, Department of Biological Education, 42075, Konya, Turkey

**^*^**Corresponding authors:

*E-mail address:* gokhanzengin@selcuk.edu.tr (Dr. Gokhan ZENGIN); [mocan.andrei@umfcluj.ro (Dr](mailto:amocanm@gmail.com(Dr). Andrei Mocan)


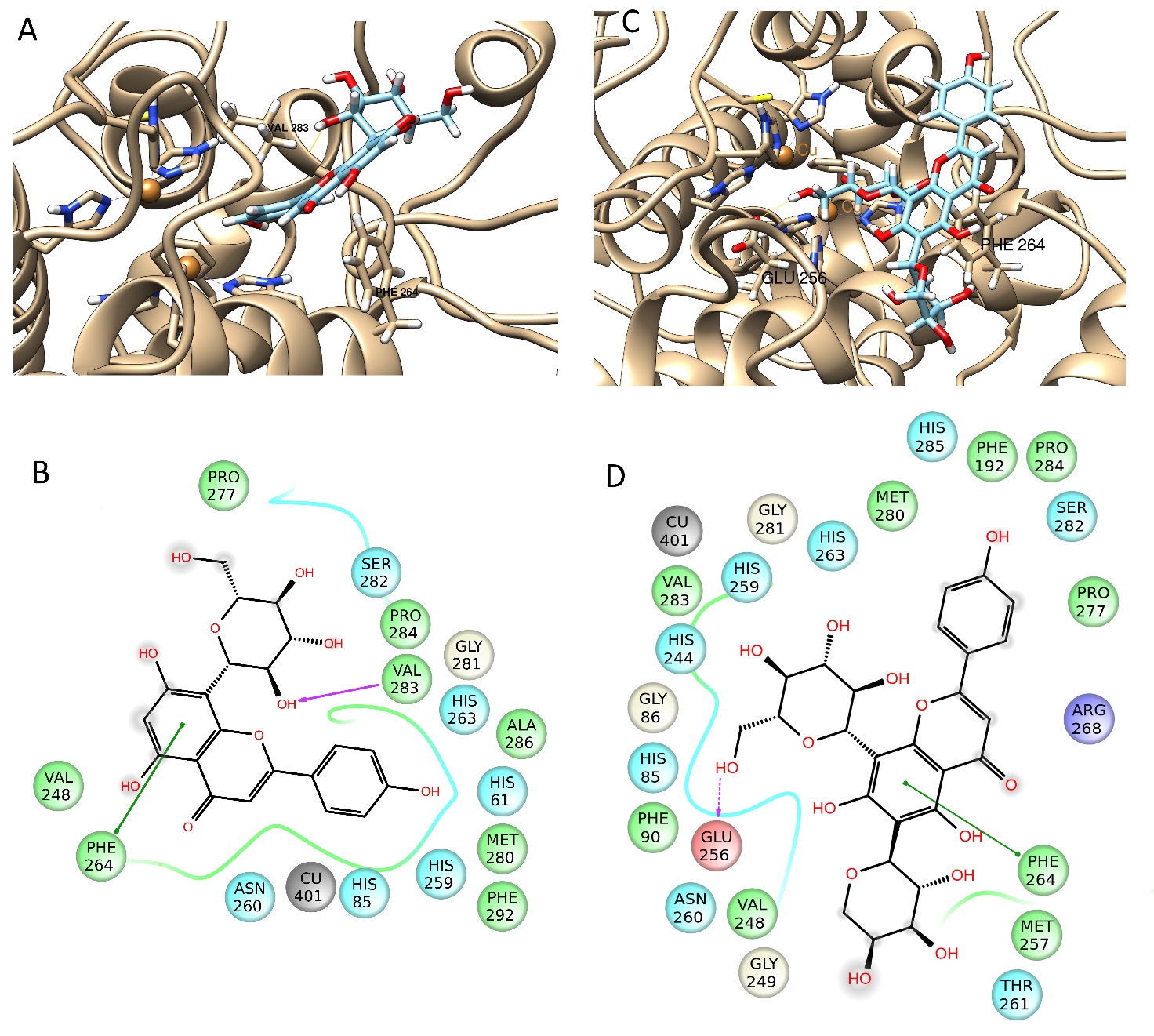


**Figure 1S**. Best pose of vitexin (A and B) and Iscoshaftoside (C and D) docked into the enzymatic pocket of tyrosinase.
